# Using HJ-CCD image and PLS algorithm to estimate the yield of field-grown winter wheat

**DOI:** 10.1038/s41598-020-62125-5

**Published:** 2020-03-20

**Authors:** Peng-Peng Zhang, Xin-Xing Zhou, Zhi-Xiang Wang, Wei Mao, Wen-Xi Li, Fei Yun, Wen-Shan Guo, Chang-Wei Tan

**Affiliations:** 1 Jiangsu Key Laboratory of Crop Genetics and Physiology/Jiangsu Co-Innovation Center for Modern Production Technology of Grain Crops/Joint International Research Laboratory of Agriculture and Agri-Product Safety of the Ministry of Education of China, Yangzhou University, Yangzhou, 225009 China; 2 Station of Land Protection of Yangzhou City, Yangzhou, 225009 China; 3 National Tobacco Cultivation and Physiology and Biochemistry Research Centre/Key Laboratory for Tobacco Cultivation of Tobacco Industry, Henan Agricultural University, Zhengzhou, 450002 China

**Keywords:** Plant ecology, Flowering

## Abstract

Remote sensing has been used as an important means of estimating crop production, especially for the estimation of crop yield in the middle and late growth period. In order to further improve the accuracy of estimating winter wheat yield through remote sensing, this study analyzed the quantitative relationship between satellite remote sensing variables obtained from HJ-CCD images and the winter wheat yield, and used the partial least square (PLS) algorithm to construct and validate the multivariate remote sensing models of estimating the yield. The research showed a close relationship between yield and most remote sensing variables. Significant multiple correlations were also recorded between most remote sensing variables. The optimal principal components numbers of PLS models used to estimate yield were 4. Green normalized difference vegetation index (GNDVI), optimized soil-adjusted vegetation index (OSAVI), normalized difference vegetation index (NDVI) and plant senescence reflectance index (PSRI) were sensitive variables for yield remote sensing estimation. Through model development and model validation evaluation, the yield estimation model’s coefficients of determination (R^2^) were 0.81 and 0.74 respectively. The root mean square error (RMSE) were 693.9 kg ha^−1^ and 786.5 kg ha^−1^. It showed that the PLS algorithm model estimates the yield better than the linear regression (LR) and principal components analysis (PCA) algorithms. The estimation accuracy was improved by more than 20% than the LR algorithm, and was 13% higher than the PCA algorithm. The results could provide an effective way to improve the estimation accuracy of winter wheat yield by remote sensing, and was conducive to large-area application and promotion.

## Introduction

Scientifically and accurately estimating crop yield is of significant importance for formulating plans for social and economic development, determining agricultural products import and export plans, ensuring national food security, guiding and regulating macroscopic planting structure, as well as improving the management skills of relevant agriculture-related enterprises and farmers^1–6^. With the improvement of spatial, temporal and spectral resolutions of remote sensing data and the significant reduction of cost, currently remote sensing has been widely used in the estimation of production of all kinds of food crops, and it has become a research focus in the interdisciplinary field combining remote sensing and agriculture^7^.

At present, there were many methods and means for estimating crop yield, such as crop yield meteorological forecast, artificial sampling survey, statistical simulation model, remote sensing estimation and so on^8,9^. Using a Criteria/Wofost simulation model that included the new numerical scheme for soil water balance, some researchers compared field data collected at the university of bologna’s experimental farm in 1977–1987 with the median wheat yield, and the predicted value was consistent with the observed value^10^. Other researches have suggested that the mars-crop yield forecasting system (M-CYFS) model was more consistent as a predictor of crop yield than meteorological predictors since these predictors summarize the succession of agrometeorological conditions for the yield of the entire growing season^11^. By using the environmental policy integrated climate (EPIC) crop growth model and daily standardized precipitation evapotranspiration index (SPEI), a comprehensive method to quantitatively evaluate the effects of drought on crop yield was proposed^12^. Among them, the crop yield meteorological forecast was suitable for small-area crops production prediction, but for large-area crops, due to large variations in field meteorological conditions in different wheat areas, the estimation accuracy was low. Manual sampling surveys had higher precision, but were time-consuming and labor intensive, and the cost was too high for large scale application. The statistical simulation model was a non-subjective method for constructing a mathematical relationship model based on historical data and estimating the future trend, but may result in errors due to climate change^13^. Remote sensing has been a high-tech method of obtaining large-area, fast, dynamic and multi-scale information on farmland. It has great application potential in large-scale crop growth monitoring, crop yield estimation, agricultural monitoring and forecasting, and agricultural resources survey^14–16^. In China and abroad, considerable work has been done on remote sensing estimation of crops, and great progress has been made^17,18^. Based on moderate-resolution imaging spectroradiometer (MODIS) derived normalized difference vegetation index (NDVI) data, a global agricultural monitoring system for crop monitoring and yield forecasting was built^19^. Based on advance very high resolution radiometer (AVHRR) data, the yield prediction model established by principal component analysis (PCA) had an estimated yield error of less than 8%^20^. The satellite-based vegetation index could be used to predict wheat yield six weeks before the time of harvest in Punjab province of Pakistan^21^. Through the data were obtained on time series remote sensing images fused with high temporal and spatial resolutions, along with grain yield and protein contents at maturity, preliminary harvest was showed that filling and anthesis stages were the best time to estimate wheat yield^22^. Some research showed that both agroclimate + MODIS-NDVI and agroclimate + MODIS-environmental vegetation index (EVI) performed equally well predicting spring wheat yield at the ecodistrict scale^23^. Through using NDVI derived from the data of the MODIS, the method for estimating and forecasting wheat yield in Hungary in the period of 2003–2015 was improved and obtained better prediction results^24^. In evaluating the influencing factors of wheat yield of the four populations, partial least square (PLS) algorithm could reveal the control factors on wheat yield in the study area and provided a reference tool for analyses in other crops or areas^25^. However, the satellite remote sensing data used in these researches was relatively short-lived, and the stability of the model simulation effect needed further testing. Some researchers argued that they lacked the spatial detail necessary for studying vegetation phenology in heterogeneous landscapes while MODIS and AVHRR have been the sensors most often used in remote sensing based phenological analysis^26^. Relevant research summarized yield estimation methods in each region through remote sensing and illustrated the importance of distinguishing between accuracy for spatial and temporal variation^27^. The data sources of the earth observation satellites were chiefly low spatial resolution MODIS, national oceanic and atmospheric administration (NOAA)/AVHRR images^23,28^, medium spatial resolution India remote-sensing satellite (IRS-P6), enhanced thematic mapper (ETM), thematic mapper (TM) images, and high spatial resolution Quickbird, SPOT, IKONOS, ALOS foreign images^29–31^. However, these data sources were expensive, which limited their use in small and medium research units and production management departments^32,33^.Therefore, it was of great significance to promote the application of image data obtained by satellites developed by China in remote sensing of agricultural conditions. On September 6, 2008, China successfully launched satellites A and B (abbreviated as HJ-CCD) of the “Environment and Disaster Monitoring and Forecasting Small Satellite Constellation System” with independent intellectual property rights. The satellites were equipped with wide-band CCD sensors with spatial resolution of the sensor being 30 m. The resolution was 2 d when satellites A and B were making observations simultaneously, making them an ideal data source for agricultural remote sensing operation.

The objectives of the present study were to investigate the quantitative relationship between the yield and satellite remote sensing variables during flowering period, and developed an effective way to improve the estimation accuracy of winter wheat yield by remote sensing.

## Results and analysis

### Yield distribution

The amplitude of variation, mean, standard deviation and standard error of the model development and validation were similar (Table 1). At the same time, the model development and model validation samples had desirable consistency.

**Table 1:** Distribution of the yield in the model development and model validation (yield unit: kg ha^-1^).

Sample set	Number of samples	Amplitude of variation	Mean	Standard deviation	Standard error
Model development	159	3053.98 ~ 9566.56	5292.51	1314.53	104.25
Model validation	106	4444.82 ~ 9852.93	7115.77	1191.43	115.72

### Quantitative analysis between remote sensing variables and yield

The quantitative analysis of the yield and remote sensing variables of 159 samples in the model development showed that there were significant or extremely significant relationships between the yield and most remote sensing variables (Table 2). The yield was most closely related to structure intensive pigment index (PSRI), followed by green normalized difference vegetation index (GNDVI), the correlation coefficients being −0.69 and −0.65, respectively. A large proportion of correlations between the yield and vegetation indices were obviously better than single bands. Most remote sensing variables had considerable multiple pairwise correlations, where the correlation coefficients were almost between 0.80 and 1.00. In particular, single-band B_1_–B_4_ pairwise correlation coefficients were between 0.95 and 0.99, and the pairwise correlation coefficient of most vegetation indices was above 0.90. It indicated that the model established by PLS algorithm was more reasonable than the traditional statistical algorithms and the ordinary least squares method, and might lead to better results.

**Table 2:** Correlation between remote sensing variables and winter wheat yield (n = 159).

	Yield	B_1_	B_2_	B_3_	B_4_	NDVI	SAVI	OSAVI	NRI	GNDVI	SIPI	PSRI	DVI	RVI
Yield	1.00													
B_1_	−0.51	1.00												
B_2_	−0.47	0.99	1.00											
B_3_	−0.33	0.99	0.99	1.00										
B_4_	−0.29	0.96	0.98	0.95	1.00									
NDVI	−0.61	0.86	0.85	0.85	0.97	1.00								
SAVI	−0.49	0.86	0.88	0.85	0.95	0.99	1.00							
OSAVI	−0.48	0.87	0.90	0.84	0.97	0.99	1.00	1.00						
NRI	0.11	0.96	0.93	0.96	0.83	0.80	0.78	0.73	1.00					
GNDVI	−0.65	0.94	0.95	0.93	0.97	0.98	0.95	0.98	0.91	1.00				
SIPI	−0.54	0.94	0.94	0.92	0.97	0.98	0.95	0.95	0.84	0.99	1.00			
PSRI	−0.69	0.95	0.96	0.91	0.93	0.86	0.86	0.87	0.93	0.93	0.97	1.00		
DVI	−0.22	0.81	0.93	0.88	0.97	0.99	0.98	0.97	0.81	0.98	0.98	0.91	1.00	
RVI	−0.23	0.82	0.81	0.80	0.80	0.96	0.98	0.96	0.65	0.91	0.91	0.69	0.97	1.00

B1, B2, B3 and B4 denoted spectrum reflectance at blue, green, red and near infrared bands, respectively.

### PLS model

**Figure 1:**
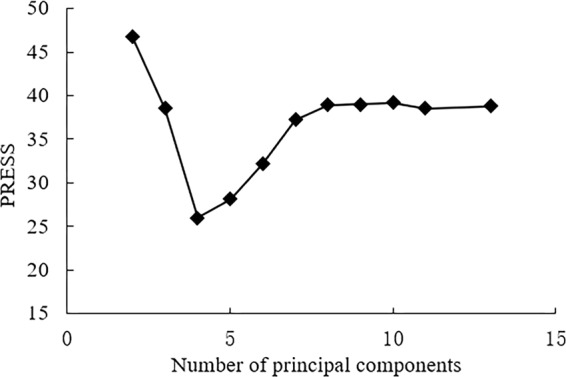
PRESS changes with the principal components.

According to the predictive residual error sum of square (PRESS) minimum value, the number of optimal principal components could be determined. Figure 1 showed the variation of PRESS with the number of principal components obtained from the yield’s model development. At the beginning, as the number of principal components increased, the yield’s PRESS value decreased to a large extent. It has indicated that due to the small number of principal components, the model fitting was extremely inadequate. It meant that the missing fitting phenomenon occurred. When the principal components numbers of the yield’s model was 4, the PRESS value (25.96) was the smallest. After that, as the number of principal components increased, the yield’s PRESS value increased sharply, until it tended to be saturated. Via this, it was indicated that the over-fitting phenomenon occurred due to too many principal components. Therefore, it was reasonable to select the number of principal components corresponding to the minimum PRESS value. Therefore, the optimal principal components numbers of the yield models based on PLS algorithm was 4.

Based on the PLS algorithm, the yield model had the four vegetation indices with the principal components number of 4, namely, GNDVI, optimized soil-adjusted vegetation index (OSAVI), NDVI and PSRI as independent variables, and the yield was the dependent variable. The yield estimation model was constructed by the yield model development and the HJ-CCD images during the three periods 2016-05-02, 2017-04-24, and 2018-04-26 was:

1 $${\rm{Yield}}=2011.7\times {\rm{GNDVI}}+1673.7\times {\rm{OSAVI}}+1821.4\times {\rm{NDVI}}-2103.8\times {\rm{PSRI}}+2810.2$$

**Figure 2:**
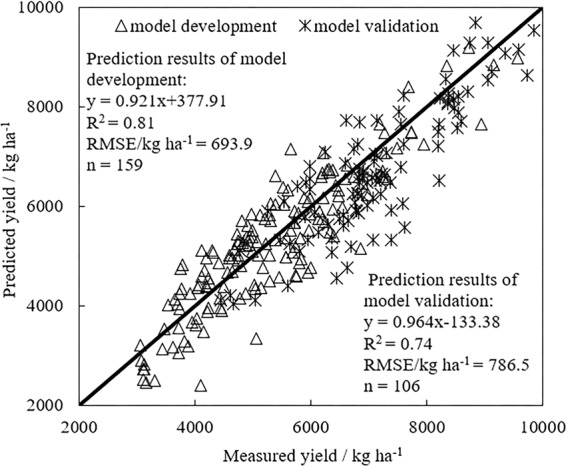
Evaluation of the yield model.

The optimal linear regression equation and its coefficient of determination (R^2^) and root mean square error (RMSE) were obtained. Figure 2 showed the evaluation of the yield model’s estimation ability. It could be seen from Fig. 2 that the model development samples number was larger than the model validation samples number. The R^2^ of the linear equation established by the model development was significantly larger than R^2^ of the model validation. The model development RMSE was significantly smaller than the model validation RMSE. It indicated that the prediction model effect of the model development samples was significantly better than the model validation. Thereby, it has theoretically conformed to the model’s estimation law. In addition, The R^2^ values between the predicted and measured yield were greater than 0.7 and the RMSE were 693.9 kg ha^−1^ and 786.5 kg ha^−1^, respectively. These results indicated that the PLS model could be used effectively to estimate the winter wheat yield.

In order to compare with the traditional algorithms, the linear regression (LR) and PCA algorithms were used to establish the yield’s estimation model through the model development and model validation samples, respectively. The model of the predicted and measured values of the yield were evaluated by R^2^ and RMSE. The specific process was not described here. Table 3 showed the comparison of estimation results with PLS, LR and PCA based on the model development and model validation. It showed that the sample number was the same. The PLS algorithm models’ R^2^ of the yield were greater than LR and PCA algorithms models, and RMSE were smaller than the LR and PCA algorithms models. This indicated that the PLS algorithm model was better than the LR and PCA algorithms in estimating the yield. The estimation accuracies based on the yield model development and model validation were 19.68% and 25.73% higher than LR algorithm models, respectively, and were 13.49% and 12.86% higher than PCA algorithm models, respectively. The estimation accuracies were above 90%.

**Table 3:** Comparison of predicted results with PLS, LR and PCA.

Algorithm	Number of principal components	Number of samples		R^2^		RMSE/kg ha^−1^		Accuracy/%	
		Model development	Model validation	Model development	Model validation	Model development	Model validation	Model development	Model validation
PLS	4	159	106	0.81	0.74	693.9	786.5	92.43	90.38
PCA	5	159	106	0.63	0.56	1054.7	1067.3	78.94	77.52
LR	0	159	106	0.57	0.47	1123.6	1342.7	72.75	64.65

**Figure 3:**
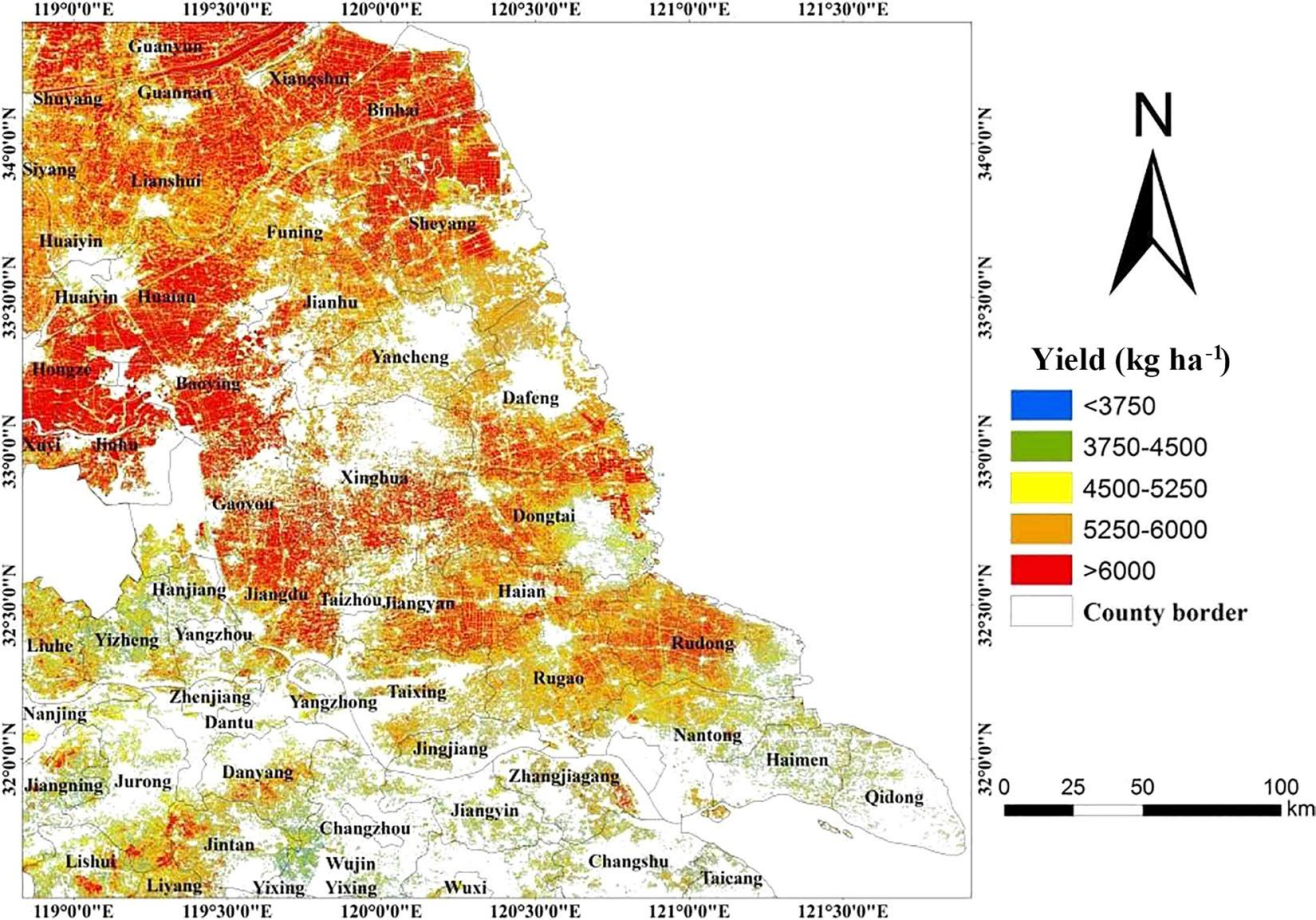
Spatial distribution of winter wheat yield in central Jiangsu region, China.

According to the above analyses, GNDVI, NDVI, PSRI and OSAVI maps were generated using 2018-04-26 HJ-CCD images. On those the winter wheat planting data was superimposed to remove the non-winter wheat area by one-to-one solution and binarization mask. Based on the administrative boundary vector data, as well as the above PLS model, the spatial distribution map of estimating winter wheat yield in central Jiangsu was produced (Fig. 3). The distribution of the yield was mainly higher than 5250 kg ha^−1^, of which Yancheng and its surrounding wheat areas was mainly 4500–6000 kg ha^−1^ and the northern wheat area of Jiangyan was more than 6000 kg ha^−1^. The number in the south wheat area rarely appeared above 6000 kg ha^−1^, and the Yangtze River area was mainly 3750–5250 kg ha^−1^, especially the south of the Yangtze River, which was mainly 3750–4500 kg ha^−1^. By predicting the results, relevant departments and farmers can formulate corresponding management and trade policies in advance, so as to achieve the effects of graded harvest and quantitative purchasing and storage. Remote sensing technology can be used to monitor crops in a wide range of areas so that agricultural management and farmers can obtain timely crop yield information. This technology saves the cost of manpower and material resources to the greatest extent and has great scientific and production significance.

## Discussions

At present, the remote sensing images used in the crop estimation were mainly originated via MODIS, NOAA/AVHRR, etc.^23,28^. These images had low spatial resolution and were difficult to apply to high-precision winter wheat remote sensing estimation in small areas. On the other hand, the high-resolution images such as Quickbird, SPOT, IKONOS were costly^30,31^. The medium-resolution TM images had revisiting periods of 16 days, making it difficult to obtain high-quality data in time. This limited continuous crop monitoring and made it inappropriate to predict crop yield^34^. The HJ-CCD satellites developed by China have been put into use one after another. The quality of the data obtained was continuously improved and was provided free of charge to users. This has created a convenient data platform for remote sensing and estimation of regional crop’s quality and productivity^35^. The experimental area of the present research has been located in the coastal area along the Yangtze River in Jiangsu Province. The whole wheat field has been fragmented and as a result the planting structure was complex. The time resolution of the selected HJ-CCD image was 2d, and the scanning width of the single scene image was 750 km. These characteristics could meet the estimation demands for the actual regional winter wheat. Considering time resolution, spatial resolution and cost, the HJ-CCD image was more appropriate than the data of MODIS, TM, Quickbird, etc.

There was a close relationship between yield and most remote sensing variables. In addition, there were considerable multiple correlations between most remote sensing variables. This made it difficult to establish a higher precision remote sensing estimation model of the yield using traditional algorithms^6,36–38^. In this study, the PLS algorithm was used to construct the remote sensing estimation model of the yield with GNDVI, OSAVI, NDVI and PSRI as the independent variables. The correlation between the yield and these remote sensing variables was extremely significant. They could be easily extracted and calculated from the HJ-CCD image. The RMSE values of the yield’s estimation model based on these remote sensing variables as the independent variables were lower than the traditional LR and PCA models. The results showed that the PLS model, as a new multivariate analysis method, had a very high adaptability in yield estimation, especially when there were many variables and multiple correlations in the analysis. The PLS algorithm could effectively optimize the dependent variables, and its model was significantly better than LR and PCA algorithms in the yield’s estimation of winter wheat. The effects of crop spectral parameters and remote sensing vegetation index on crops are not often single. It is often multiple remote sensing variables acting on a single agronomic index. Therefore, based on the traditional univariate model and linear regression algorithm, it is difficult to make a more accurate prediction. PLS algorithm is highly adaptive to the condition of multivariate and high correlation. The results were consistent with Hanen *et al*.^39^ and Zhao *et al*.^40^. Based on the traditional linear regression algorithm, it is easy to build a model for monitoring and predicting crop quality, growth and yield, but the prediction accuracy is often not up to the requirements. For the prediction of agricultural indicators under the control of multiple variables, the PLS algorithm is superior to the general linear regression model. The results of this research better than Liu *et al*.^41^ and Xue *et al*.^42^. In order to reflect it in a better way, the actual situation of field planting and different varieties were selected in the experiment. Using the data derived from different varieties as test samples, the results were of more general in significance. It was helpful to the promotion and application in actual production. It indicated that it was feasible to use the PLS model to estimate winter wheat yield with high precision. It has, therefore, provided an effective method and technical support for the high-precision estimation of aerospace remote sensing images, and was also conducive to large-area application and promotion.

According to the spatial distribution map for predicting winter wheat yield in Jiangsu province (Fig. 3). Northern Jiangsu, especially in the northwest of Gaoyou and Sheyang county, the yield is higher than other areas. The yield of the middle region of Jiangsu is relatively lower than that of the northern region, which basically maintains around 5000–6000 kg ha^−1^. But along the river and the region south of the Yangtze river, the yield is generally low. There was large scale wheat cultivation in northern Jiangsu. Local agricultural facilities were well developed, and agricultural production was mainly in the form of farms for planting and management. Therefore, winter wheat planting could be managed uniformly, with good cultivation measures and maximum implementation. Overall agricultural development and management in the middle area of Jiangsu was slightly worse than that in north of Jiangsu. But the whole structure of agricultural facilities and agricultural management could meet the planting of winter wheat. Therefore, wheat yield presented a general level range. The southern Jiangsu area was mostly metropolis and urban area with less farmland, and there were few areas for wheat cultivation. At the same time, the local farmland was chaotic and scattered, mainly operated by small farmer households. It might result in good cultivation measures and management could not be used effectively. Therefore, the winter wheat yield in southern Jiangsu was relatively low. The predicted results of the spatial distribution map for predicting winter wheat yield in Jiangsu province were basically consistent with the actual situation of winter wheat production. It indicated that it was feasible to use the PLS model to predict winter wheat yield with high precision. It has, therefore, provided an effective method and technical support for the high precision remote sensing prediction of winter wheat yield.

The samples used in the research were relatively concentrated, basically ranging from 4000 kg ha^−1^ to 9000 kg ha^−1^. Samples with higher or lower content were relatively few, showing above 9000 kg ha^−1^ and less than 4000 kg ha^−1^. There was a lack of samples more than 10063 kg ha^−1^ and less than 2152 kg ha^−1^. If the variation of the yield samples was increased, the PLS model would be further optimized and its application range would be further expanded. The remote sensing estimation models of the yield would become more reliable. The results obtained were based only on the HJ-CCD data of the Jiangsu experimental area. Therefore, whether the model would be applicable to other remote sensing sensor data and/or estimate the winter wheat yield in other areas needed further study.

The present study did not compare the PLS algorithm with artificial neural network (ANN)^43,44^, support vector machines (SVM)^45^, geostatistics^46^, etc. Simultaneously, it also did not take into account the factors affecting winter wheat cultivation. These algorithms and factors actually had a wide range of influence on the estimation results of winter wheat yield and needed further study.

## Conclusions

In the present research, a close relationship between yield and most remote sensing variables were found. Significant multiple correlations were also obtained between most remote sensing variables. GNDVI, OSAVI, NDVI and PSRI were sensitive for remotely estimating the yield. Through the model development and model validation evaluation, the estimation model of the yield had R^2^ of 0.81 and 0.74, and the RMSE were 693.9 kg ha^−1^ and 786.5 kg ha^−1^. It showed that the PLS algorithm estimated the yield better than the LR and PCA algorithms. The improvements were by more than 20% than the LR algorithm and more than 13% higher than the PCA algorithm. The PLS model provided an effective way to improve the accuracy of estimating winter wheat yield through remote sensing.

## Materials and methods

### Test design and data acquisition

For the present investigation, data collection was carried out in 5 counties, namely, Taixing, Jiangyan, Yizheng, Xinghua and Dafeng in Jiangsu Province in 2016, the People’s Republic of China. There were 15–20 sampling points in each county, totaling 92. The location of each sampling site was determined by using a Juno ST hand-held GPS meter (Trimble Co. USA). The survey mainly included information collection on winter wheat varieties, growth period, population growth and disasters status (mainly pests and diseases). Winter wheat varieties were of medium and weak gluten type, mainly *Yangmai* 1*3*, *Yangmai 15*, *Yangmai 16* and *Yangfumai 2*. These varieties were available in the experimental counties. GPS was used to locate the positions during the mature stage. Samples were taken back to measure the yields in the laboratory.

**Figure 4:**
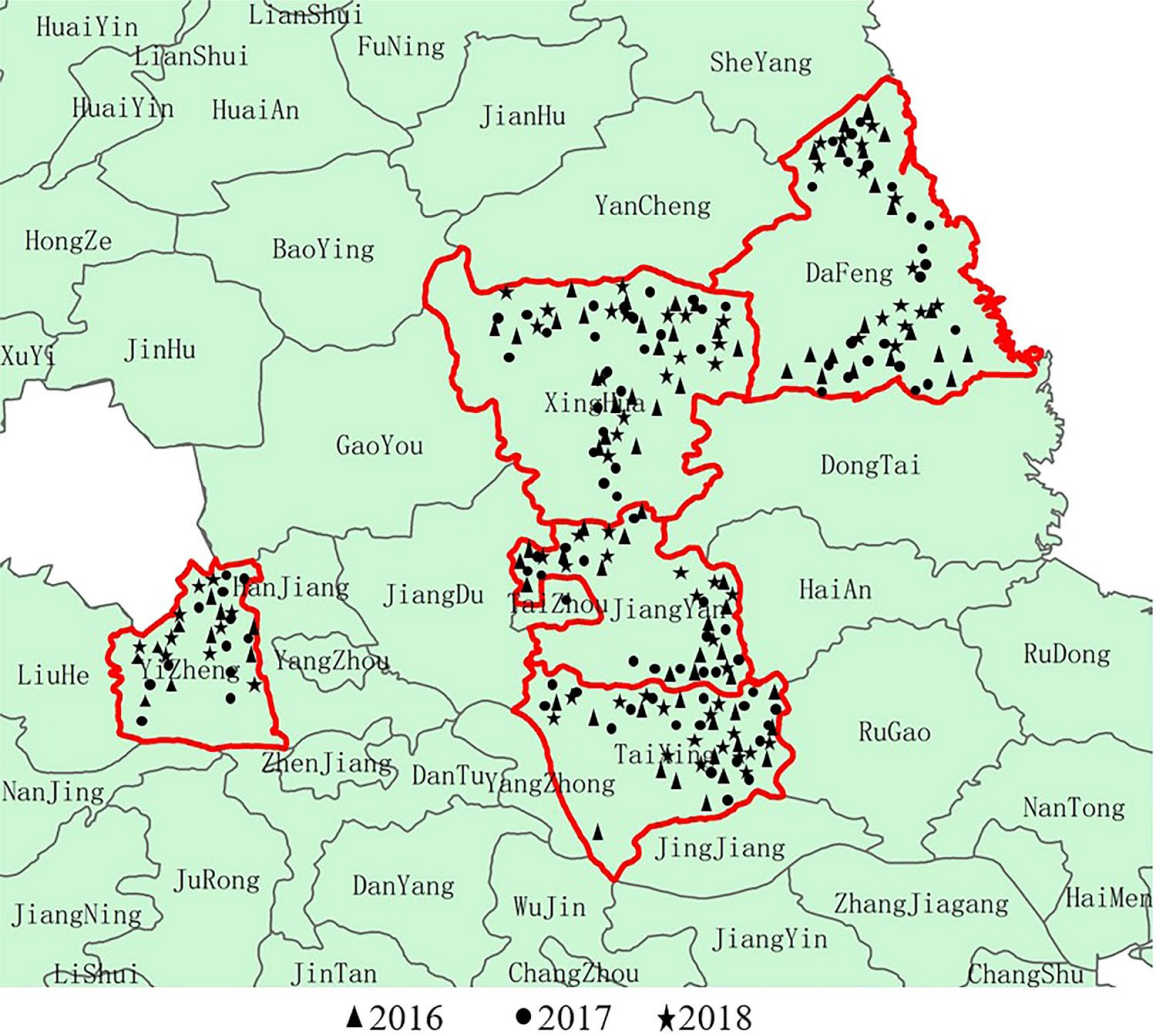
Sampling point information for three consecutive years.

A total of 3 tests were launched in the experimental counties from 2016–2018 to collect data. The satellite data was HJ-CCD images taken at flowering stage of winter wheat. Data collection for Test 1, 2 and 3 were conducted on May 2, 2016; April 24, 2017 and April 26, 2018, respectively. The sampling points considered for the Test 1–3 were 92, 96 and 67, respectively. Figure 4 showed the distribution of sampling points in 2016, 2017 and 2018.

The yield data measured in Tests 1–3 were arranged in the order of the yield values in the winter wheat grain sample. In order to enhance the stability of the estimation model, the numerical samples of 265 yields were randomly divided into model development and model validation according to a ratio of 3:2, on the premise that the maximum and minimum yields were placed in the modeling sample set.

### Image preprocessing

Environment for Visualizing Images (ENVI 5.4) software was used to preprocess satellite images. Firstly, georeferencing process was performed. The specific operation was that the 1:100,000 topographic maps of Jiangsu area were used to roughly correct the satellite image. Thereafter, the GPS control points for ground measuring were used to precisely correct the satellite image. This helped to ensure that the precision of geometric correction was better than one pixel. Atmospheric correction and reflectance conversion were carried out by empirical linear method^14,47^. According to the analysis of the results, the corresponding single-band value graph was obtained by using a workflow called band math in ENVI. Data of wheat growing areas were obtained by supervised classification. The winter wheat planting data were superimposed and the non-winter wheat area was eliminated by one-to-one solution and binarization mask. By using the administrative boundary vector data and the above PLS model, the spatial distribution map of winter wheat yield in Jiangsu province was produced.

### Yield measurement

**Figure 5:**
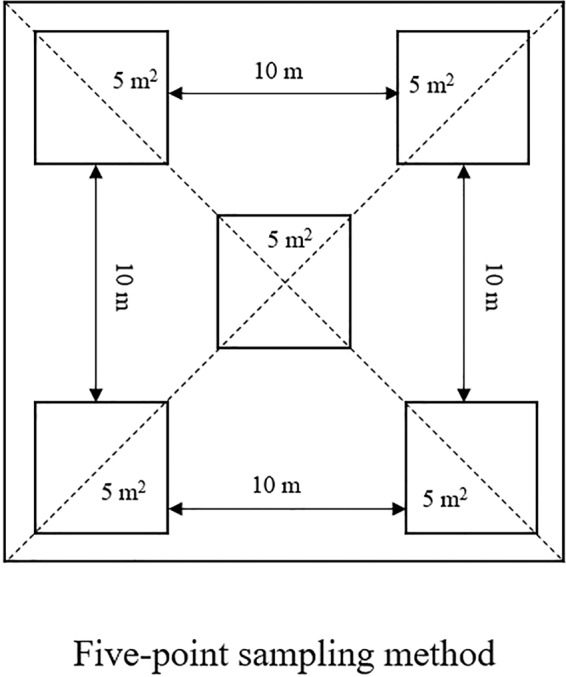
Five-point sampling method.

During the maturity period, 5 plots were selected by five-point sampling method (Fig. 5) in the middle part in the field. The four plots on the periphery formed a rectangle, and they were 10m apart. Each plot was 5m^2^, and each plot grew evenly, which could represent the overall situation of the field. And the field area sampled should be more than 2 hectares. The grains of the five plots were brought back to the laboratory. All the samples from five plots were shelled and weighed separately. After averaging the yields of these 5 plots, the average value was the value of the sampling site and was converted to the value of one hectare, which was the yield.

### Satellite remote sensing variables

In combination with the physical significance of spectral indices, selection of model parameters was based on the spectral characteristics of crops and the available literatures in home and abroad. In this study, four HJ-CCD bands and nine common spectral vegetation indices were selected (Table 4) as independent variables for PLS analysis in order to construct remote sensing estimation model of the yield.

**Table 4:** Formulas of remote sensing vegetation indices.

Vegetation index	Abbreviation	Algorithm	Source
Normalized difference vegetation index	NDVI	(B_4_−B_3_)/(B_4_+B_3_)	^52^
Soil-adjusted vegetation index	SAVI	(B_4_−B_3_) / (B_4_+B_3_+0.5)*1.5	^53^
Optimized soil-adjusted vegetation index	OSAVI	(B_4_−B_3_) /(B_4_+B_3_+0.16)*1.16	^54^
Nitrogen reflectance index	NRI	(B_2_−B_3_)/(B_2_+B_3_)	^55^
Green normalized difference vegetation index	GNDVI	(B_4_−B_2_)/(B_4_+B_2_)	^56^
Structure intensive pigment index	SIPI	(B_4_−B_1_)/(B_4_+B_1_)	^57^
Plant senescence reflectance index	PSRI	(B_3_−B_1_)/B_4_	^55^
Difference vegetation index	DVI	B_4_ − B_3_	^58^
Ratio vegetation index	RVI	B_4_ / B_3_	^59^

To extract spectral band brightness values of corresponding GPS positioning sampling points, ENVI 5.4 and geographic information system software (ArcGIS 10.2) were used. actual these satellite remote sensing variables were calculated using Excel 2016.

### PLS regression

PLS regression was first applied to the field of chemometrics. PLS regression has been considered as a new multivariate analysis method with wide applicability. The PLS regression was concentrated on the characteristics of principal component, linear regression and typical multiple regression analysis. It could effectively solve many problems. Such as, problems that could not be solved by ordinary multiple regression, especially when there were many variables and multiple correlations. In these cases, PLS could effectively decompose and screen the comprehensive variables that were most explanatory to the dependent variables. Therefore, the established model was more reliable than the ordinary regression analysis. The PLS method first extracted a new variable called component as an independent variable, and established a linear combination relationship between the dependent variable and the independent variable. The coefficient was determined by PLS calculation, and then the regression equation of the dependent variable was constructed. The regression model established by the PLS method could be expressed by Eq. (2):

2 $${y}_{m}={a}_{0m}+{a}_{1m}{x}_{1}+\ldots +{a}_{Pm}{x}_{p}\,(m=1,2,\ldots p)$$

where *x*_*1*_*, ···, x*_*p*_ were linear combinations of remote sensing variables, *a*_*0m*_*, a*_*1m*_*, ···, a*_*pm*_ were parameters of the regression model and could be computed by PLS.

When the model was established by PLS algorithm, the increase of the number of principal components would improve the accuracy of the model. But too many principal components would cause over-fitting and the error would increase. Therefore, it was very important to determine the optimal principal components number of the PLS model. In this study, the sum of squared residuals was calculated by the cross-validation method. The smaller the PRESS value, the stronger the estimation ability of the model. Therefore, the optimal principal components number could be determined according to the minimum value of PRESS. PRESS could be expressed by Eq. (3):

3 $$PRESS=\mathop{\sum }\limits_{i=1}^{k}{({y}_{i}-{y}_{i,-i})}^{2}$$

where *y*_*i*_*, y*_*i,-i*_ were the measured value corresponding to the *i*th sample and the estimated value when the *i*th sample was excluded, and *k* was the number of validating iterations.

For the basic principles and specific practices of the PLS algorithm and PRESS, please refer to references^25,48^, which were not described here. Both the PLS and PRESS processes were performed by a self-written MATLAB program. In this study, the yields were estimated based on the PLS algorithm. Then it was compared with the yields’ estimation model based on LR and PCA algorithms. For the explanation of LR and PCA algorithms, please refer to the references^49,50^, which were not described here.

### Evaluation of the model

Using the samples of the model development and model validation, the model was evaluated by plotting the 1:1 relationship graph between the predicted and measured values of the yield. The evaluation indices were the R^2^ and the RMSE^51^. On one hand, the larger the R^2^, the better the model is. On the other hand, the smaller the RMSE, the stronger the estimation ability of the model is. RMSE and estimation accuracy were calculated using Eqs. (4) and (5), respectively:

4 $$RMSE=\sqrt{\frac{1}{n}\mathop{\sum }\limits_{i=1}^{n}{({y}_{i}-{\hat{y}}_{i})}^{2}}$$

5 $$Accuray=\frac{1}{n}\mathop{\sum }\limits_{i=1}^{k}|{y}_{i}-{\hat{y}}_{i}|$$

where *y*_*i*_ and ${\hat{y}}_{i}$ represented measured values and predicted values of wheat yields, respectively, and *n* was the number of samples.
